# Marine Bacterial Exopolymers-Mediated Green Synthesis of Noble Metal Nanoparticles with Antimicrobial Properties

**DOI:** 10.3390/polym11071157

**Published:** 2019-07-07

**Authors:** Angela Scala, Anna Piperno, Alexandru Hada, Simion Astilean, Adriana Vulpoi, Giovanna Ginestra, Andreana Marino, Antonia Nostro, Vincenzo Zammuto, Concetta Gugliandolo

**Affiliations:** 1 Dipartimento di Scienze Chimiche, Biologiche, Farmaceutiche ed Ambientali, Università di Messina, Viale F. Stagno d’Alcontres 31, 98166 Messina, Italy; 2 Nanobiophotonics Center, Interdisciplinary Research Institute in Bio-Nano-Sciences and Faculty of Physics, Babes-Bolyai University, T Laurian 42, 400271 Cluj-Napoca, Romania; 3 Nanostructured Materials and Bio-Nano-Interfaces Center, Interdisciplinary Research Institute in BioNano-Sciences, Babes-Bolyai University, T. Laurian 42, 400271 Cluj-Napoca, Romania

**Keywords:** EPS, Au nanoparticles, Ag nanoparticles, AgCl nanoparticles, *S. aureus*, *E. coli*, *P. aeruginosa*, *C. albicans*

## Abstract

A straightforward and green method for the synthesis of gold, silver, and silver chloride nanoparticles (Au NPs and Ag/AgCl NPs) was developed using three different microbial exopolymers (EP) as reducing and stabilizing agents. The exopolysaccharides **EPS B3-15** and **EPS T14** and the poly-**γ**-glutamic acid **γ-PGA-APA** were produced by thermophilic bacteria isolated from shallow hydrothermal vents off the Eolian Islands (Italy) in the Mediterranean Sea. The production of metal NPs was monitored by UV−Vis measurements by the typical plasmon resonance absorption peak and their antimicrobial activity towards Gram-positive and Gram- negative bacteria (*Staphylococcus aureus,* *Escherichia coli* and *Pseudomonas aeruginosa*), as well as fungi (*Candida albicans*) was investigated. The biological evaluation showed no activity for EP-Au NPs, except against *E. coli*, whereas EP-Ag NPs exhibited a broad-spectrum of activity. The chemical composition, morphology, and size of EP-Ag NPs were investigated by UV–Vis, zeta potential (ζ), dynamic light scattering (DLS) measurements and transmission electron microscopy (TEM). The best antimicrobial results were obtained for **EPS B3-15-Ag NPs** and **EPS T14-Ag NPs** (Minimum Inhibitory Concentration, MIC: 9.37–45 µg/mL; Minimum Bactericidal Concentration/Minimum Fungicidal Concentration, MBC/MFC: 11.25–75 µg/mL).

## 1. Introduction

Noble metal nanoparticles, particularly gold and silver (Au NPs and Ag NPs, respectively), have attracted increasing interest due to their potential applications in several fields ranging from catalysis, sensors, optoelectronics, biotechnology to medicine [1]. They possess a unique combination of advantageous physicochemical features, different from those of the bulk metal, such as high surface-to-volume ratio, versatile surface functionalization, surface plasmon resonance properties, making them suitable to be used in advanced application fields [2]. A variety of biomedical implementations/exploitations of noble metal NPs, including hyperthermia therapy, theranostics, and drug delivery, was investigated [3]. Moreover, Ag NPs also possess antimicrobial properties, as they are effective in inhibiting growth of Gram-positive and Gram-negative bacteria [4].

The synthesis of Au NPs and Ag NPs is generally carried out by chemical reduction of metal precursors (HAuCl_4_ and AgNO_3_, respectively), in solution, in the presence of reductants (typically borohydride, ascorbate, citrate, or elemental hydrogen) and different organic capping agents, such as surfactants, polymers, solid supports, or ligands with functional groups suitable for stabilization, in order to prevent unwanted aggregation of the colloids [5,6]. Additionally, Au NPs and Ag NPs can be advantageously synthesized by an eco-friendly laser ablation method, using a pulsed laser beam to ablate a metal target immersed in a solvent, without reducing and capping agents [7,8]. Recently, a straightforward and environmentally benign method for the synthesis of Au NPs using aqueous solutions of amino acids, HAuCl_4_, and white light (xenon lamp) was reported [9]. Light irradiation acted as catalyst for the oxidation of the amino acids, which resulted in metal reduction (photooxidation/reduction). No additives, such as surfactants or reducing agents, were used.

Nowadays, the development of reliable and eco-friendly procedures for the synthesis of metal nanoparticles and nanocomposites is crucial for successful advancement in nanotechnology applications. The integration of the principles of green chemistry to nanotechnology plays a relevant role in nanoscience, as green synthesis could assure a sustainable, safe, and cost-effective production of NPs [10] by the use of renewable sources, non-hazardous materials, safer solvents, the prevention of waste production, and the reduction in the number of synthetic steps.

The concept of green NPs preparation was first proposed by Raveendran et al., who utilized glucose as a reductant and starch as a capping agent to prepare silver NPs [11].

To date, several approaches have been reported regarding the use of environmentally friendly materials for the synthesis of metal NPs, in particular plant extracts [12], fungi [13], bacteria, and enzymes [14]. It was demonstrated that the principal biomolecules present in plant extracts responsible for the reduction of metal ions to form NPs are polyphenols, sugars, proteins, and vitamins [15]. Notoriously, polysaccharides possess hydroxyl groups, a hemiacetal reducing end, and other functionalities that can play important roles in both reduction and the stabilization of inorganic NPs [16]. A range of structurally diverse polysaccharides are synthesized by bacteria of all taxa, included marine bacteria [17,18], and secreted into the external environment, referred to as “exopolysaccharides” (EPS) [19].

To date, different bacterial EPS have been used as reducing and capping agent for the preparation of Au NPs [20,21,22,23,24], Ag NPs [16,25] and also silver chloride nanoparticles (AgCl NPs) [25,26,27].

We explore herein the potential of three different marine microbial exopolymers (EP), produced by thermophilic bacteria isolated from shallow hydrothermal vents off the Eolian Island (Italy), to be used as template for the nucleation and stabilization of noble metal NPs. The EP were selected on the basis of their unique chemical composition and their physicochemical and biological properties [28,29]. They could be useful to respond to the increasing demand of new active bioproducts for biotechnological purposes.

In particular, for the environmentally friendly production of Au NPs and Ag/AgCl NPs, we selected EPS produced by *Bacillus licheniformis* strain B3-15 (**EPS B3-15**) [30] and by the thermophilic *Bacillus licheniformis* T14 (**EPS T14**) [31], as well as the poly-γ-glutamic acids (**γ**–PGA) produced by *Bacillus horneckiae* strain APA (**γ-PGA-APA**) [32].

The aim of our work was to explore green and mild conditions for the preparation of EP- stabilized noble metal NPs (EP-Au NPs and EP-Ag NPs) and to investigate their antimicrobial properties against Gram-positive (*Staphylococcus aureus*) and Gram-negative (*Escherichia coli*, *Pseudomonas aeruginosa*) bacteria along with fungi (*Candida albicans*). A broad-spectrum of antimicrobial activity was detected for EP-Ag NPs, whereas EP-Au NPs were active only against the Gram-negative *E. coli.* The correlation among antimicrobial activity, chemical composition, and morphology of EP-Ag NPs was investigated.

The EP-mediated production of Au NPs and Ag/AgCl NPs was confirmed by UV–Vis spectroscopy, monitoring the typical Plasmon resonance absorption peak (i.e., Au NPs: ~550 nm; Ag NPs: 390–420 nm; AgCl NPs: ~250 nm). The morphology, size, and metal NPs distribution of EP-Ag NPs were investigated by UV–Vis spectroscopy, zeta potential (ζ), DLS measurements, and transmission electron microscopy (TEM).

## 2. Materials and Methods

### 2.1. Materials

Tetrachloro-auric acid (HAuCl_4_), silver nitrate (AgNO_3_), and ultrapure water were purchased from Sigma-Aldrich (Milan, Italy) and used as received. Microbial culture media were purchased from Oxoid (Milan, Italy). The exopolysaccharides **EPS B3-15** from *Bacillus licheniformis* strain B3-15, **EPS T14** from *Bacillus licheniformis* strain T14, and the poly-γ-glutamic acid (**γ-PGA-APA**) from *Bacillus horneckiae* strain APA, were previously described [30,31,32]. Briefly, the strains were isolated from thermal fluid samples collected from shallow hydrothermal vents off the Eolian Islands (Italy) (Table 1). The **EPS B3-15** (Mw 600 KDa) contained carbohydrates (66%) and proteins (5.0%), monosaccharide composition of mannose and glucose, tetrasaccharide repeating units, and a manno- pyranosidic anomeric configuration. The **EPS T14** (Mw 1000 kDa) contained carbohydrates (70%), proteins (0.6%), nucleic acids (<1%), and uronic acids (1.3%). Its main purified fraction was composed of fructose, fucose, and glucose, as well as galactosamine and mannose as contaminants, trisaccharide repeating units, and a β-manno-pyranosidic anomeric configuration. The **γ-PGA-APA** (Mw 890–1000 kDa) contained a low percentage of carbohydrates (14%), proteins (1.8%), nucleic acids (<1%), and uronic acids (0.3%).

### 2.2. Preparation of EP-Au NPs

One mg of HAuCl_4_ was dissolved in 10 mL of H_2_O and heated to boiling. Then a solution containing 2 mg of EP dissolved in 1 mL of H_2_O was added. The mixture was kept under stirring at 100 °C. The progress of the reaction was monitored via UV−Vis measurement by the increase of intensity of the typical plasmon resonance absorption peak of Au NPs at ~550 nm. The formation of Au NPs was completed within 3 h with **EPS B3-15** and after 1 h and 30 min for **γ-PGA-APA**, whereas the plasmon resonance absorption peak was not detectable for **EPS T14**. The EP-Au NPs solutions were kept at 4 °C for 12 h and then used for the biological assays. EP-Au NPs were tested in the range of metal concentration from 45 µg/mL to 0.04 µg/mL.

### 2.3. Preparation of EP-Ag NPs

A solution of 10 mL of EP (1 mg/mL) was prepared and mixed with 1 mL of AgNO_3_ solution (1 mg/mL) and 0.5 mL of NaCl solution (0.5 mg/mL). The mixture was left under magnetic stirring at room temperature (rt = 25 °C). The progress of the reaction was monitored by UV−Vis absorption spectra, following the increase of intensity of the plasmon resonance absorption peak of Ag NPs in the range 390–420 nm and of AgCl NPs at ~250 nm. The reduction of Ag^+^ was completed within 2 days for **EPS B3-15** and **EPS T14**. The experiment with **γ-PGA-APA** failed. The EP-Ag NPs were used for the biological assays either as freshly prepared or as reconstituted solutions from lyophilized samples. Freshly prepared EP-Ag NPs were tested in the range of metal concentration from 45 µg/mL to 0.04 µg/mL. Lyophilized EP-Ag NPs were tested in the range of metal concentration from 150 µg/mL to 0.15 µg/mL.

### 2.4. Preparation of γ-PGA-APA-Ag NPs

For the preparation of **γ-PGA-APA-Ag NPs**, the method proposed by Selvin et al. was used [23], with slight modifications. Briefly, a solution of 10 mL of EP (1 mg/mL) was prepared and mixed with 1 mL of AgNO_3_ solution (1 mg/mL) and 0.5 mL of NaCl solution (0.5 mg/mL). The mixture was left under magnetic stirring at room temperature (rt = 25 °C) for 24 h. Afterwards, 0.2 mL of NaBH_4_ solution (5 mg/mL) was added and the mixture left under magnetic stirring for 2 h. The resulting solution was centrifuged at 6000 rpm for 10 min, and then the supernatant was filtered through a 0.45 μm pore size filter. The solutions were dialyzed in a dialysis bag (MW cutoff 500 Da) against deionized water for 24 h. Finally, the dialyzed solutions were lyophilized to obtain the **γ-PGA-APA-Ag NPs**.

### 2.5. Nanoparticle Characterization

UV-Visible spectra were recorded on a 1 nm spectral resolution spectrophotometer Jasco V-670 (Pfungstadt, Germany) in the range 200–800 nm. Transmission electron microscopy (TEM) measurements were performed with the high-resolution microscope TecnaiG2 F20 X-TWIN (FEI Company, Hillsboro, OR, USA). Zeta potential (ζ) and dynamic light scattering (DLS) measurements were carried out with a Malvern Zetasizer Nano ZS-900 (Malvern, UK) equipped with a 633 nm He-Ne laser.

### 2.6. Antimicrobial Activity

*Staphylococcus aureus* ATCC 6538, *Escherichia coli* ATCC 10536, *Pseudomonas aeruginosa* ATCC 9027, and *Candida albicans* ATCC 10,231 were selected for this study. Microbial cultures were grown in Mueller–Hinton Broth (bacteria) and Sabouraud Dextrose Broth (*C. albicans*) at 37 °C for 24 h. The Minimum Inhibitory Concentration (MIC) of each sample was determined using a broth dilution micro-method in 96-wells polystyrene microtitre plates, according to the Clinical and Laboratory Standards Institute (CLSI 2009) guidelines, with some modifications [33]. The MIC was considered as the lowest concentration of each sample giving a complete growth inhibition in comparison with a growth control. To determine Minimum Bactericidal Concentration (MBC) or Minimum Fungicidal Concentration (MFC), aliquots (20 µL) from each clear well were spot-inoculated on Mueller–Hinton Agar or Sabouraud Dextrose Agar, respectively. MBC/MFC was defined as the lowest concentration of sample that allowed no microbial growth after incubation at 37 °C for 24–48 h. All the determinations were performed in triplicate. EP and AgNO_3_ were used as controls in the range of concentration from 500 μg/mL to 0.49 μg/mL.

## 3. Results and Discussion

### 3.1. Synthesis and Characterization of EP-Au NPs

The use of polysaccharides that are rich in hydroxyl groups and other functionalities as the reductant and the stabilizer for the synthesis of noble metal NPs is considered to be an eco-friendly alternative to the traditional chemical-reducing method. Herein, we performed the green synthesis of Au NPs and Ag/AgCl NPs using marine bacterial exopolymers, **EPS B3-15, EPS T14**, and **γ-PGA-APA** as reducing and capping agent. Different experimental conditions, such as temperature, time, and metal ions concentration, were investigated to set up the best mild protocol.

**Figure 1:**
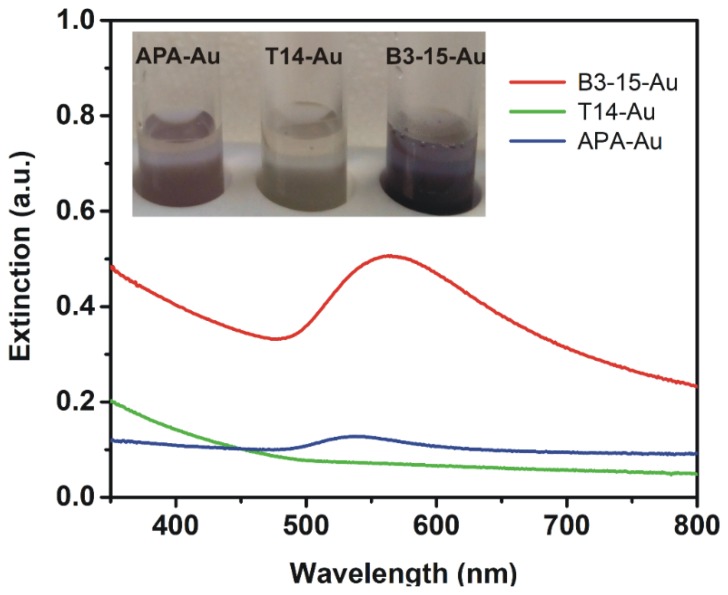
Extinction spectra of **EPS B3-15-Au NPs** (red), **EPS T14-Au NPs** (green), and **γ-PGA-APA-Au NPs** (blue) together with photographs of the three samples.

The synthetic approach for the production of Au NPs consists of the addition of an aqueous solution of EP (2 mg/mL) to a boiling aqueous solution of tetrachloro-auric(III) acid (1 mg/10 mL). When EP was added into the yellow HAuCl_4_ solution at 100 °C, the solution slowly began to change from colorless to purple (Figure 1), suggesting that Au^3+^ was reduced to Au NPs. The EP-mediated production of Au NPs was completed at 100 °C within 3 h for **EPS B3-15** and after 1 h and 30 min for **γ-PGA-APA**, as demonstrated by UV–Vis analysis monitoring the typical plasmon resonance absorption peak of Au NPs (Figure 1). The absorption band was detected at 565 nm for **EPS B3-15-Au NPs** and at 538 nm for **γ-PGA-APA-Au NPs**, whereas it was not detected in the experiment with **EPS T14**, whose color remained almost unchanged (Figure 1).

Exploring different experimental conditions, temperature, and Au^3+^ concentration emerged as critical parameters for the Au NPs formation and we found that the best conditions are an initial concentration of Au^3+^ of 0.1 mg/mL and a temperature of 100 °C.

At room temperature (rt = 25 °C), the synthesis of Au NPs did not work at all at the Au^3+^ concentrations tested (0.1 mg/mL and 0.05 mg/mL). Non-optimal Au NPs were produced at temperatures below 60 °C and at low gold ions concentration (i.e., 0.05 mg/mL). Moreover, no color change and plasmon absorbance were detected in blank experiments in the absence of EP.

### 3.2. Synthesis and Characterization of EP-Ag NPs

Silver NPs were obtained via in situ reduction of Ag^+^ under the macromolecular environment of the EP. The synthesis was performed using AgNO_3_ in the presence of NaCl, resulting in the formation of both silver and silver chloride nanoparticles (Ag/AgCl NPs). In fact, along with Ag NPs, AgCl NPs are produced as a result of the reaction between NaCl and AgNO_3_. Rasulov et al., reported that formation of Ag/AgCl NPs on a polysaccharide matrix in the presence of chlorides is a complex process [25], in which the interaction of silver and chloride ions in a polysaccharide matrix resulted first in the formation of AgCl NPs, while the synthesis of the Ag NPs started only when the chloride ions were consumed.

**Figure 2:**
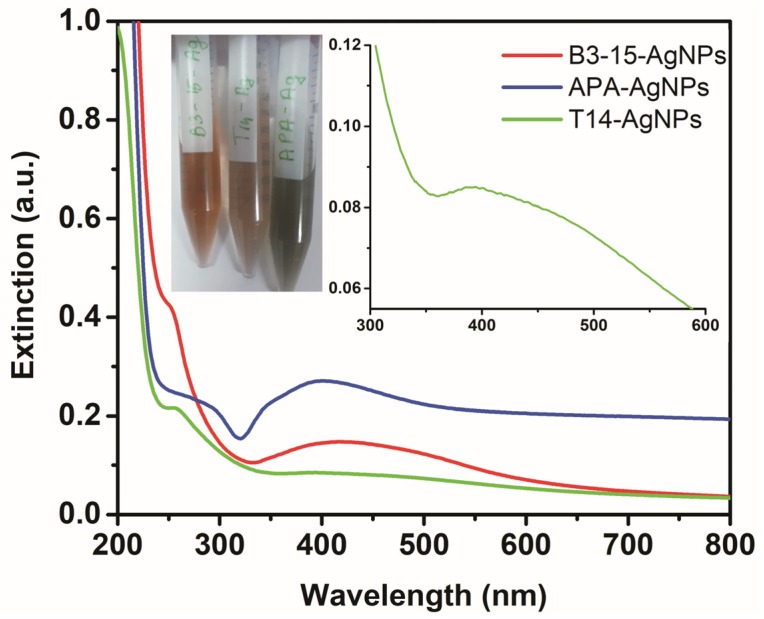
Extinction spectra of **EPS B3-15-Ag NPs** (red), **γ-PGA-APA-Ag NPs** (blue) and **EPS T14- Ag NPs** (green), with photographs of the three samples. The inset reports a magnification of the band at 394 nm of **EPS T14-Ag NPs**.

The synthetic method for the production of Ag/AgCl NPs consisted of the addition of an AgNO_3_ solution into the EP aqueous solution, followed by the addition of NaCl. The reduction of Ag^+^ was completed within two days for **EPS B3-15** and **EPS T14**. The reduction process could be monitored by the color change of the solution, as the synthesis of silver NPs results in the formation of a brownish color (Figure 2), and by UV–Vis analysis monitoring the typical plasmon resonance absorption peak of silver NPs (Figure 2). The colloidal solutions had absorption peaks at ~250 nm and in the range 390–420 nm, confirming the formation of AgCl NPs and Ag NPs, respectively. In particular, **EPS B3-15-Ag NPs** and **EPS T14-Ag NPs** exhibited similar maximum absorption peaks at 251 and 252 nm respectively, ascribable to the formation of AgCl NPs, in good agreement with the literature data [25], whereas the peaks at 421 and 394 nm, respectively, were attributed to the Ag NPs absorption. The intensity of the band at 421 nm (**EPS B3-15-Ag NPs**) and 394 nm (**EPS T14-Ag NPs**) indicated a different Ag/AgCl NPs ratio, using **EPS B3-15** and **EPS T14**.

The green protocol failed using **γ-PGA-APA** which was unable to reduce AgNO_3_. An alternative method was applied for the preparation of **γ-PGA-APA-Ag NPs**, using NaBH_4_ as reducing agent, leading to the exclusive formation of Ag NPs with a maximum absorption peak at 401 nm.

**Figure 3:**
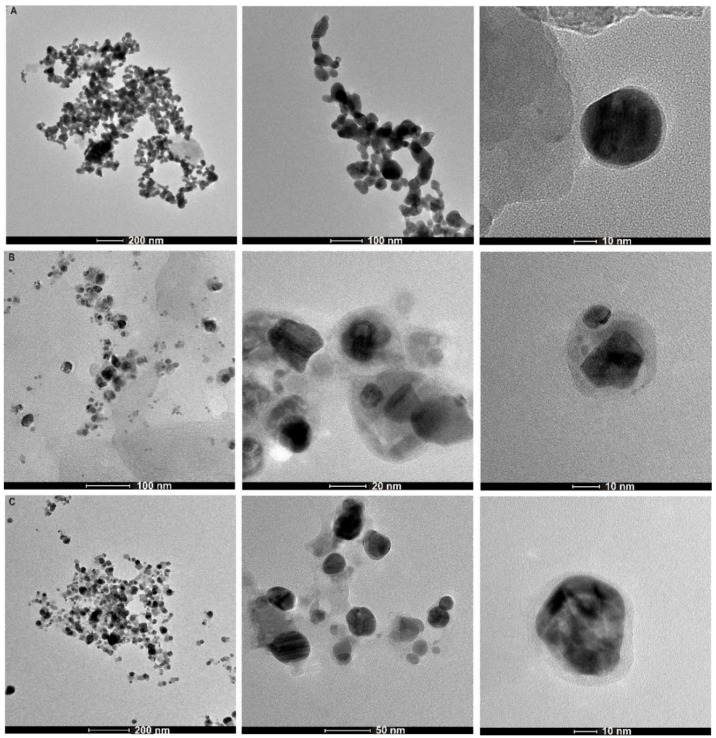
TEM micrographs of **γ-PGA-APA-Ag NPs** (**A**), **EPS B3-15-Ag NPs** (**B**), **EPS T14-Ag NPs** (**C**) at different magnifications.

The morphology of the **γ-PGA-APA-Ag NPs**, **EPS B3-15-Ag NPs**, and **EPS T14-Ag NPs** was investigated by TEM analyses (Figure 3). TEM images of **γ-PGA-APA-Ag NPs** (Figure 3A) showed a well-defined and uniform spherical structure with an average diameter of 35 nm. The relative magnified image revealed the presence a polymeric external layer with a thickness of about 2–3 nm. In contrast, **EPS B3-15-Ag NPs** are irregular in shape and dimension and they are enveloped in a large polymeric vesicle (Figure 3B). **EPS T14-Ag NPs** are sphere-like in structure with an average diameter of 40 nm and the relative magnified image showed a significant external polymeric layer with a thickness of about 6–7 nm (Figure 3C).

The obtained results suggested that the EP composition affecting the features of both the silver NPs and the external capping. **γ-PGA-APA** was unable to form silver NPs by the green protocol and only a small polymeric vesicle was detected around Ag NPs produced in the presence of NaBH_4_. Conversely, **EPS B3-15** and **EPS T14** showed a good capability to act as reducing and capping agents during the production of Ag/AgCl NPs and in the latter case the NPs were incorporated in a large polymeric vesicle.

We assumed that the sugar component of EP was essential for the reduction process; in fact, the carbohydrate content of **EPS B3-15** and **EPS T14** (66% and 70%, respectively) [30,31] assured the reduction of AgNO_3_, whereas the lower content of carbohydrates in **γ-PGA-APA** (i.e., 14%) [32] was inadequate to induce the reduction of silver ions.

The zeta potential values of **γ-PGA-APA-Ag NPs**, **EPS B3-15-Ag NPs** and **EPS T14-Ag NPs** were –25.4, –32.6, and –20.2 mV, respectively, indicating a good stability of the colloidal solutions.

Their size was investigated by DLS measurements, that revealed the formation of agglomerate nanoparticles with agglomerate diameters in the micrometer range for **γ-PGA-APA-Ag NPs** (≈1170 nm) and **EPS T14-Ag NPs** (≈1827 nm), whereas for **EPS B3-15-Ag NPs** a more dispersed material having a diameter in the nanometer range was detected (average diameter ≈170 nm). In comparison to TEM analysis, DLS measured the hydrodynamic diameter of the particles, which included hydration layer and polymer shells, leading, in general, to a larger particle size. Moreover, DLS is unable to resolve polydisperse samples and in the samples with multimodal distribution, the scattered light of the larger particles or agglomerates strongly overlaid that of the smaller particles [34,35]. Severe artefacts can be produced by DLS since large particles scatter the light much more intensely than smaller particles, producing strongly misleading results in particle samples with different sizes and shapes. We supposed that our polymeric capping layer, determining the formation of NPs and/or aggregates with different size, shape and distribution, could have affected the DLS results. In fact, according to other literature data [34,36], we found a significant difference between DLS and TEM sizes measurements attributed not only to the fundamental difference between the dry and the hydrodynamic radius of particles, but also to the sample features of our EPS-stabilized Ag NPs. Considering the difference between DLS and TEM sizes measurements, DLS data were not further investigated due to the sample-specific limitations of that analysis.

### 3.3. Antimicrobial Activity

Noble metal NPs are known to be efficient biocidal agents [27,37]. The antibacterial and antifungal activity of EP-Au NPs and EP-Ag NPs was first investigated against selected Gram-positive (*S. aureus*), Gram-negative (*E. coli*) bacterial strains and against fungi (*C. albicans*). EP-Au NPs and EP-Ag NPs were tested in the range of metal concentration from 45 µg/mL to 0.04 µg/mL. In addition, the more active samples were selected to investigate their activity against *P. aeruginosa*. All the results of antimicrobial activity are reported in Table 2. The NPs obtained by **EPS T14** and **EPS B3-15** were active in contrast with the samples obtained by **γ-PGA-APA**, which showed only a moderate effect against *E. coli*.

The **EPS T14-Ag NPs** and **EPS B3-15-Ag NPs** demonstrated a broader activity with respect to the corresponding gold NPs. Notably, they showed a good activity against all microorganisms tested (MIC and MBC: 11.25–45 µg/mL), comparable to that of the positive control AgNO_3_ (MIC and MBC: 7.81–31.25 µg/mL), and, between them, the best efficacy was detected for **EPS B3-15-Ag NPs**. Interestingly, they were bactericidal against Gram-negative strains, including *P. aeruginosa* strain. Otherwise, although **EPS T14-Au NPs** and **EPS B3-15-Au NPs** showed a good activity versus the *E. coli* strain (MIC: 22.5 µg/mL), they were not selected for further investigations due to their limited antimicrobial spectrum.

The EP used as control, without any metal NPs (i.e., **γ-PGA-APA**, **EPS T14**, **EPS B3-15**) showed no activity even if at the highest tested concentration.

We assumed that the broad-spectrum antimicrobial activity of EP-Ag NPs could be correlated to the presence of both Ag NPs and AgCl NPs, as demonstrated by either the inactivity of **γ-PGA-APA-Ag NPs** (which contains only Ag NPs) and by the best result obtained with **EPS B3-15-Ag NPs** containing both types of silver NPs (see Figure 2 and relative discussion).

Interestingly, the antimicrobial activity of **EPS T14-Ag NPs** and **EPS B3-15-Ag NPs** was also maintained after lyophilization and redispersion in water (Table 3), showing that EP-Ag NPs can be conveniently stored as lyophilized powder. Lyophilized EP-Ag NPs were tested in the range of metal concentration from 150 µg/mL to 0.15 µg/mL. These higher tested concentrations also allowed the MBC against *S. aureus* to be determined (Table 3). Freeze-drying could assure the long-term stability of colloidal nanoparticles, as the poor stability in aqueous medium of these systems is a drawback to their use [38].

## 4. Conclusions

The present study demonstrates the applicability of three different exopolymers produced from marine bacteria for the production and stabilization of Au NPs and Ag/AgCl NPs. TEM measurement carried out on EP-Ag NPs demonstrated that the nanoparticles’ size and shape differed depending on the EP used. Moreover, it was found that the chemical composition (Ag/AgCl NPs), strictly dependent of the EP nature, affected the antimicrobial activity of EP-Ag NPs. The best biological results were obtained with B3-15-Ag NPs, likely due to the presence of both Ag NPs and AgCl NPs. The antimicrobial investigation showed a broad-spectrum activity of silver NPs obtained by thermostable EPS T14 and EPS B3-15, with a bactericidal effect against all tested microorganisms, including *P. aeruginosa*. In perspective, these EPS can be successfully utilized for biotechnological, nanotechnological, and material science applications even at high temperatures.

## Figures and Tables

**Table 1:** Physicochemical characteristics of thermal fluids emitted from the shallow hydrothermal vents off the Eolian Islands and related isolated strains.

Site	Depth (m)	T (°C)	pH	Conductivity (mS/cm^−1^)	Strain	References
Bottaro, Panarea Island	8.0	55	5.4	42.9	T14	[31]
Campo 7, Panarea Island	21.3	60	4.9	49.2	APA	[32]
Porto Levante, Vulcano Island	0.7	70	5.2	-	B3-15	[30]

**Table 2:** Antimicrobial activity of EP-Au NPs and EP-Ag NPs (μg/mL).

Samples	*S. aureus*		*E. coli*		*P. aeruginosa*		*C. albicans*	
	MIC ^a^	MBC ^b^	MIC	MBC	MIC	MBC	MIC	MFC ^c^
AgNO_3_	31.25	31.25	15.62	15.62	7.81	7.81	15.62	125
γ-PGA-APA	- ^d^	-	-	-	n.d. ^e^	n.d.	-	-
EPS T14	-	-	-	-	-	-	-	-
EPS B3-15	-	-	-	-	-	-	-	-
γ-PGA-APA-Au	-	-	45	-	n.d.	n.d.	-	-
EPS T14-Au	-	-	22.5	-	n.d.	n.d.	-	-
EPS B3-15-Au	-	-	22.5	-	n.d.	n.d.	-	-
γ-PGA-APA-Ag	-	-	-	-	n.d.	n.d.	-	-
EPS T14-Ag	45	-	11.25	11.25	22.5	22.5	11.25	45
EPS B3-15-Ag	22.5	22.5	11.25	11.25	11.25	11.25	11.25	45

^a^ MIC: Minimum Inhibitory Concentration. ^b^ MBC: Minimum Bactericidal Concentration. ^c^ MFC: Minimum Fungicidal Concentration. ^d^ No activity at the highest concentration tested. ^e^ n.d. = not determined.

**Table 3:** Antimicrobial activity of lyophilized EPS T14-Ag NPs and EPS B3-15-Ag NPs (μg/mL).

Samples	*S. aureus*		*E. coli*		*C. albicans*	
	MIC	MBC	MIC	MBC	MIC	MFC
EPS T14-Ag *_lyophil._*	37.5	75	18.75	18.75	9.37	37.5
EPS B3-15-Ag *_lyophil._*	18.75	37.5	18.75	18.75	9.37	37.5
